# The Role of Thioredoxin/Peroxiredoxin in the β-Cell Defense Against Oxidative Damage

**DOI:** 10.3389/fendo.2021.718235

**Published:** 2021-09-07

**Authors:** Jennifer S. Stancill, John A. Corbett

**Affiliations:** Department of Biochemistry, Medical College of Wisconsin, Milwaukee, WI, United States

**Keywords:** peroxiredoxin, thioredoxin, oxidative stress, beta-cell, antioxidant, thioredoxin reductase

## Abstract

Oxidative stress is hypothesized to play a role in pancreatic β-cell damage, potentially contributing to β-cell dysfunction and death in both type 1 and type 2 diabetes. Oxidative stress arises when naturally occurring reactive oxygen species (ROS) are produced at levels that overwhelm the antioxidant capacity of the cell. ROS, including superoxide and hydrogen peroxide, are primarily produced by electron leak during mitochondrial oxidative metabolism. Additionally, peroxynitrite, an oxidant generated by the reaction of superoxide and nitric oxide, may also cause β-cell damage during autoimmune destruction of these cells. β-cells are thought to be susceptible to oxidative damage based on reports that they express low levels of antioxidant enzymes compared to other tissues. Furthermore, markers of oxidative damage are observed in islets from diabetic rodent models and human patients. However, recent studies have demonstrated high expression of various isoforms of peroxiredoxins, thioredoxin, and thioredoxin reductase in β-cells and have provided experimental evidence supporting a role for these enzymes in promoting β-cell function and survival in response to a variety of oxidative stressors. This mini-review will focus on the mechanism by which thioredoxins and peroxiredoxins detoxify ROS and on the protective roles of these enzymes in β-cells. Additionally, we speculate about the role of this antioxidant system in promoting insulin secretion.

## Introduction

Diabetes mellitus, affecting 29.1 million people in the United States (1), is a group of diseases characterized by high blood glucose, or hyperglycemia, and is caused by failure of pancreatic β-cells to secrete sufficient insulin to match the requirements of the body. Type 1 diabetes (T1D), making up about 5% of all diabetes cases (1), occurs when β-cells are selectively destroyed by an autoimmune process. Type 2 diabetes (T2D), making up 90-95% of all cases (1), develops in a progressive manner due to the inability of β-cells to produce enough insulin to meet the need brought on by age, inactivity, obesity, and/or genetic risk factors. In addition, approximately 10% of T2D patients 35 years of age or older develop latent autoimmune diabetes in adults (LADA), often necessitating exogenous insulin treatment (2, 3).

Oxidative stress, which occurs when there is an imbalance between generation and removal of reactive oxygen species (ROS), may contribute to β-cell damage during either T1D or T2D (4). ROS, like superoxide and hydrogen peroxide, cause oxidation of lipids, proteins, and DNA, and may eventually lead to cell death. Indeed, numerous studies have reported increased oxidative damage in islets from diabetic rodents and patients (5–13). Furthermore, β-cells are considered vulnerable to oxidative stress due to decreased expression of antioxidants, including catalase and glutathione peroxidases, compared to levels found in liver and kidney (14–17). However, recent studies have determined that one class of antioxidants called peroxiredoxins is expressed in β-cells and functions to protect them from a variety of oxidative insults. Here, we provide a review of what is known about the roles of peroxiredoxins and their partners, thioredoxin and thioredoxin reductase, in the β-cell antioxidant defense. We also speculate about how these cells may utilize peroxiredoxin redox relays, in which oxidative equivalents are transferred from peroxiredoxin to a redox-regulated target protein, to promote their function. While not discussed here, other antioxidants, including glutathione peroxidase 1 and superoxide dismutase, have also been shown to support β-cell survival, suggesting that these cells likely utilize multiple pathways to protect themselves from oxidative damage (4, 18–21).

## Types of ROS and Relevance to β-Cell Damage

A critical feature of the β-cell is its ability to link changes in metabolic flux, brought on by glucose metabolism, to changes in membrane excitability and subsequent alterations in intracellular Ca^2+^ concentration ([Ca^2+^]_i_) and insulin secretion (22, 23). Glycolysis is coupled to oxidative phosphorylation such that most of the carbons in glucose are oxidized to CO_2_ on supply of the sugar substrate (24–26). This metabolic flux allows for β-cells to respond to blood glucose changes with changes in insulin secretion that are controlled by the rates of glucose sensing and oxidation (27).

Here, we will focus on three types of ROS: superoxide (O_2_^•-^), hydrogen peroxide (H_2_O_2_), and peroxynitrite (ONOO^-^). Superoxide is primarily produced in the cell as a byproduct of mitochondrial oxidative metabolism. A small percentage of electrons passing through the electron transport chain during oxidative phosphorylation “leak out” and react with oxygen to produce superoxide (28). In diabetes, when blood glucose is chronically elevated, increased oxidative phosphorylation may increase electron leak, thus increasing superoxide production in β-cells (29, 30). Indeed, stimulatory glucose concentrations increase ROS production in insulinoma cells (31, 32) and rat islets (33). Mitochondrially-derived superoxide may contribute to ROS-mediated damage induced by pro-inflammatory cytokines (34). Superoxide can also be produced *via* NADPH oxidases, which catalyze the addition of an electron (using NADPH) to molecular oxygen (35). This is another mechanism by which inflammatory cytokines may generate ROS in β-cells (36–38).

Hydrogen peroxide can be generated from superoxide by spontaneous dismutation or through a reaction catalyzed by superoxide dismutase (SOD) (39). It can then either undergo the Fenton Reaction, reacting with free iron or copper ions to form the highly reactive hydroxyl radical (•OH), or it can be reduced to water by several different antioxidants, including catalase, glutathione peroxidase, or peroxiredoxin (39). Traditionally, superoxide dismutase, catalase, and glutathione peroxidase are considered the three primary antioxidants (39). However, the relative activities of these enzymes in islets compared to the liver are about 29%, 1%, and 2%, respectively. The comparative absence of these enzymes has led to the perception that β-cells are vulnerable to oxidative damage (14, 16, 40).

Peroxynitrite (ONOO^-^) is a reactive species generated by the diffusion-controlled reaction of superoxide and nitric oxide and has been suggested to contribute to β-cell damage in response to pro-inflammatory cytokines (41, 42). However, β-cells do not generate peroxynitrite in response to cytokines (43). In most cell types, superoxide and nitric oxide work synergistically to increase cell death over levels observed in response to either radical individually (44). In contrast, when β-cells are forced to generate peroxynitrite by providing exogenous superoxide and nitric oxide, superoxide scavenges the nitric oxide, forming peroxynitrite, and attenuates nitric oxide-mediated damage, suggesting that β-cells possess a robust mechanism to protect them from the damaging effects of peroxynitrite (43, 45, 46).

## Catalytic Mechanisms and Isoforms of Peroxiredoxins

**Figure 1:**
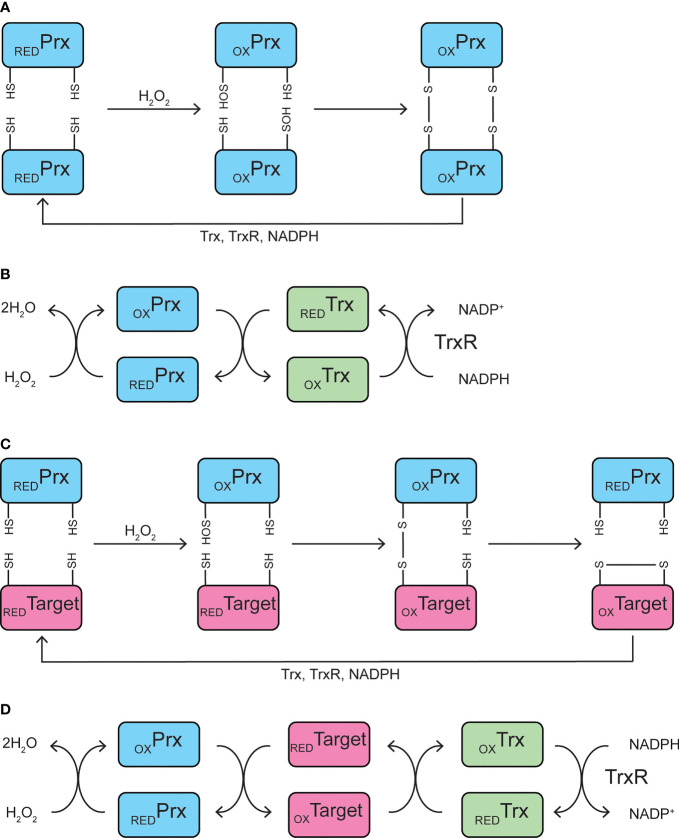
Catalytic mechanisms. **(A, B)** Peroxidase mechanism of typical 2-Cys peroxiredoxins. The peroxidatic cysteine residue of peroxiredoxin becomes oxidized to Cys-SOH upon reaction with hydrogen peroxide, which then reacts with the resolving cysteine residue on a neighboring peroxiredoxin to form an intermolecular disulfide bond **(A)**. Peroxiredoxin is oxidized by hydrogen peroxide, and is subsequently reduced by thioredoxin, which becomes oxidized itself and is reduced by thioredoxin reductase, utilizing NADPH **(B)**. **(C, D)** Potential mechanism of peroxiredoxin-mediated redox relay. The peroxidatic cysteine residue of peroxiredoxin becomes oxidized to Cys-SOH upon reaction with hydrogen peroxide, which then reacts with a redox-sensitive cysteine residue on a target protein to form a mixed disulfide intermediate. The disulfide is transferred to the target protein, resulting in an oxidized target and reduced peroxiredoxin **(C)**. Peroxiredoxin is oxidized by hydrogen peroxide and oxidizes a redox-sensitive target protein. The oxidized target protein is subsequently reduced by thioredoxin, which becomes oxidized itself and is reduced by thioredoxin reductase, utilizing NADPH **(D)**. Prx, peroxiredoxin; Trx, thioredoxin; TrxR, thioredoxin reductase; RED, reduced; OX, oxidized.

Peroxiredoxins are a class of peroxidases falling into three categories with slightly different catalytic mechanisms. Broadly, a highly conserved cysteine residue, named the “peroxidatic” Cys, is the site of oxidant reduction. Upon reaction with hydrogen peroxide, lipid peroxides, or peroxynitrite, the peroxidatic Cys becomes oxidized, forming a sulfenic acid (**Figure 1A**). The sulfenic acid then reacts with a neighboring “resolving” Cys residue of another peroxiredoxin molecule, forming a disulfide bond. This disulfide is reduced by thioredoxin, a small disulfide reductase, which was originally discovered as the reducer of ribonucleotide reductase, the enzyme that catalyzes the generation of deoxyribonucleotides from ribonucleotides and is necessary for DNA replication (47). Upon reducing the disulfide bond of peroxiredoxin, thioredoxin itself becomes oxidized, losing catalytic activity. Thioredoxin reductase utilizes electrons from NADPH to reduce thioredoxin, allowing the antioxidant cycle to continue (**Figure 1B**) (48). If the peroxide level exceeds a certain threshold, peroxiredoxins are subject to hyperoxidation, resulting in the formation of sulfinic or sulfonic acids, the latter inhibiting the enzyme (49). This feature of the peroxiredoxin catalytic mechanism becomes pertinent when remembering that it is common when examining the β-cell response to ROS to rely on a single or repeated bolus of diluted peroxide added to the cell culture medium (50). Later, we will discuss why it may be better practice to provide the oxidant using a continuous delivery method.

There are six mammalian peroxiredoxins of three different classes: typical 2-Cys, atypical 2-Cys, and 1-Cys. The catalytic mechanism of typical and atypical 2-Cys isoforms differ only in disulfide bond formation. With typical 2-Cys peroxiredoxins, the disulfide created upon peroxide reduction forms an intermolecular bond, while atypical 2-Cys peroxiredoxins utilize an intramolecular disulfide bond (49). As the name suggests, typical 2-Cys isoforms make up the largest class, with PRDX1, PRDX2, PRDX3, and PRDX4 falling into this category. PRDX5 is the only atypical 2-cys peroxiredoxin known to exist in mammals (49). The final peroxiredoxin, PRDX6, is the only known mammalian peroxiredoxin of the 1-Cys class. The catalytic mechanism of PRDX6 is different from other peroxiredoxins in that no disulfide bond is formed because there is no other nearby Cys reside available (49). Additionally, PRDX6 does not rely on reduction by thioredoxin, thioredoxin reductase, or NADPH, but instead relies on glutathione (49). Several studies have examined the potential role of PRDX6 in the β-cell defense against oxidative damage (51, 52), but because our review is focused on the peroxiredoxin/thioredoxin antioxidant system, they will not be discussed here.

## Protective Roles of Thioredoxin and Thioredoxin Reductase in β-Cells

To assess the potential role of oxidative damage in the development of either T1 or T2D, many studies have utilized models in which various antioxidant enzymes are overexpressed in β-cells or islets. In one such study, Hotta and colleagues generated a non-obese diabetic (NOD, a T1D model) transgenic mouse in which human thioredoxin was over-expressed under control of the human insulin promoter (53). Thioredoxin overexpression delays the onset of diabetes and protects against β-cell cytotoxicity induced by streptozotocin (STZ), a β-cell toxin (53). Similarly, mouse islets overexpressing human thioredoxin by lentivirus delivery bestow better glycemic control and reduce diabetic frequency when transplanted into diabetic NOD mice (54). High-fat diet-induced glucose intolerance is reduced in mice overexpressing thioredoxin in all tissues (under the control of the β-actin promoter) (55). Treatment of INS 832/13 cells, an insulinoma cell line, with small molecule thioredoxin mimetics attenuates cell death induced by treatment with the thioredoxin reductase inhibitor, auranofin (56). Interestingly, thioredoxin 1 (TRX1) has been shown to be secreted by MIN6 insulinoma cells during hypoxic conditions, and exogenously added TRX1 protects MIN6 cells from death induced by hypoxia (57). Collectively, these studies demonstrate that addition of exogenous thioredoxin, either *via* protein over-expression or use of peptide mimetics, improves β-cell survival under a variety of environmental stresses.

One molecule that has received a lot of attention in the islet field in recent decades is thioredoxin-interacting protein (TXNIP), as it was identified as the gene with the largest increase in expression in human islets exposed to high (20 mM) glucose (58). TXNIP is an endogenous thioredoxin inhibitor that was later found to be elevated in rodent models of diabetes and has therefore been associated with the β-cell response to stress (59). More recent mechanistic studies suggest that TXNIP may play a causal role in β-cell damage and death in response to hyperglycemia, as TXNIP overexpression causes β-cell apoptosis, and TXNIP deficiency promotes β-cell survival (60–62).

While many studies have examined a role for exogenous thioredoxin in protecting β-cells from damage, the finding that depletion of a protein inhibitor of thioredoxin (TXNIP) protects against β-cell death suggests that endogenous thioredoxin activity supports β-cell survival. Indeed, thioredoxin reductase inhibition by auranofin or depletion by siRNAs targeting cytoplasmic TXNRD1 sensitizes INS 832/13 cells or dispersed rat islet cells to DNA damage and death induced by hydrogen peroxide delivered continuously over time but not delivered as a one-time bolus (63). Delivery of hydrogen peroxide as a bolus addition likely overwhelms the oxidant capacity of the thioredoxin reductase antioxidant cycle since peroxiredoxins, as discussed earlier, are subject to inhibition if the oxidant exceeds a certain threshold (49). This finding emphasizes the importance of choosing an oxidant delivery method that more closely recapitulates an *in vivo* situation, such as glucose/glucose oxidase to continuously deliver hydrogen peroxide extracellularly, or a redox cycler, such as menadione, to deliver hydrogen peroxide intracellularly. Cytoplasmic TXNRD1 also protects INS 832/13 cells and rat islets from peroxynitrite-mediated DNA damage and death (64). These recent findings suggest that, while β-cells express low levels of catalase and glutathione peroxidase, they possess an antioxidant defense that requires thioredoxin and thioredoxin reductase for regeneration through reduction of oxidized peroxiredoxins. The identification of this endogenous system may have been masked by frequent bolus hydrogen peroxide delivery methods in previous studies.

## Protective Roles of Peroxiredoxins in β-Cells

Among the first to assess peroxiredoxin expression in β-cells were Bast and colleagues in 2002, who observed that PRDX1 and PRDX2 are expressed in the cytoplasm of INS-1 insulinoma cells and in islets of BalbC mice (65). Interestingly, based on immunofluorescence staining of mouse pancreatic tissue sections, peroxiredoxins are predominantly expressed in islets, with only low expression in surrounding exocrine tissue (65). Other studies have corroborated the expression of these antioxidants in β-cells or islets (63, 66, 67). Importantly, PRDX1 and PRDX2 protein expression is increased in INS-1 cells in response to a number of environmental stressors, including inflammatory cytokines (IL-1β, IFN-*γ*, and TNF-α), hydrogen peroxide delivered as a bolus, alloxan, and streptozotocin (STZ) (65). The observation that peroxiredoxin expression is responsive to agents that generate oxidative stress provides one potential explanation as to why β-cells may express peroxiredoxins and not other antioxidants, like catalase or glutathione peroxidase, as expression of these latter enzymes is not responsive to environmental stressors (40).

There is controversy concerning the responsivity of peroxiredoxins to stress, as Zhao and Wang observed decreased protein expression of PRDX2 in MIN6 insulinoma cells in response to inflammatory cytokines (IL-1β, IFN-*γ*, and TNF-α) or STZ, rather than increased expression (66). And Jin et al. observed decreased PRDX1 expression following STZ treatment of MIN6 cells, but stable PRDX2 expression (67). PRDX3, the mitochondrially-localized isoform, is also expressed in β-cells, and its expression is increased following 24-hr cytokine treatment (IL-1β, IFN-*γ*, and TNF-α) in C57/BL6 mouse islets (68). However, acute exposure of C57/BL6 mouse islets to cytokines (IL-1β and IFN-*γ*, 6 hr) does not stimulate a change the mRNA accumulation of any peroxiredoxin isoform but increases TXNRD1 expression (69).

Subsequent studies have aimed to determine the contribution of peroxiredoxins to protecting β-cells from different stimuli, with varying, and in some cases contradictory, results. Inhibition of peroxiredoxins with covalent inhibitor conoidin A increases sensitivity of INS 832/13 cells to hydrogen peroxide delivered continuously by glucose/glucose oxidase or by the redox cycler menadione, suggesting a general role for these antioxidants in protecting β-cells against oxidative stress (64). Peroxiredoxin inhibition also sensitizes INS 832/13 cells to damage and death by peroxynitrite (64). PRDX1 was determined to be the primary isoform involved in protection against hydrogen peroxide and peroxynitrite, as specific PRDX1 depletion increases INS 832/13 cell death in response to these oxidants (64). Overexpression of PRDX2, the other main cytoplasmic isoform, in MIN6 cells protects against death induced by palmitic acid, cytokines, or STZ while siRNA-mediated depletion had the opposite effect (66). However, siRNA-mediated knockdown of PRDX2 in INS 832/13 cells has no effect on damage or death induced by continuously-delivered hydrogen peroxide or peroxynitrite (64). The disagreement regarding contribution of PRDX2 to protection against oxidative damage may be explained by the difference in stimuli or in cell model utilized.

Both PRDX3, which is localized to the mitochondria, and PRDX4, the ER-localized isoform, are expressed in β-cells (63, 68, 70). In insulinoma cells, specific knockdown of PRDX3 or PRDX4 has no effect on cell viability in response to hydrogen peroxide (64, 70). This result may be attributed to the dominant antioxidant actions of the cytoplasmic peroxiredoxins. However, over expression of PRDX3 in RINm5F insulinoma cells protects against death induced by hydrogen peroxide delivered as a bolus (68). Similarly, overexpression of human PRDX4 reduces ROS generation, protects against death induced by hydrogen peroxide delivered as a bolus, and increases insulin content in INS-1E cells (70). Global overexpression of PRDX4 protects mice against STZ-mediated diabetes, and this protection is associated with reduced islet damage (71). Together, these studies suggest that cytoplasmic 2-Cys peroxiredoxin PRDX1 likely plays a primary role in protecting β-cells against oxidative damage, and that overexpression of any 2-Cys isoform imparts additional protection.

## Involvement of NADPH-Generating Enzyme G6PD

Thioredoxin reductase requires NADPH as a co-factor to reduce oxidized thioredoxin (49). In the cytoplasm, glucose-6-phosphate dehydrogenase (G6PD) catalyzes the reaction of glucose-6-phosphate to 6-phospho-gluconate, generating NADPH. G6PD deficiency sensitizes mouse embryonic stem cells to death induced by hydrogen peroxide delivered by glucose oxidase, suggesting that this enzyme is critical for the mammalian cytoplasmic antioxidant defense (72). In β-cells specifically, siRNA-mediated depletion of G6PD in MIN6 cells increases ROS accumulation, DNA fragmentation, and markers of apoptosis (73). Additionally, G6PD-deficient mice have reduced islet size compared to wildtype mice (73). Global transgenic overexpression of G6PD leads to improved glucose tolerance, increased β-cell function, and reduced pancreatic oxidative damage in mice (74). These results suggest that NADPH generated by G6PD is necessary for β-cell detoxification of ROS and for normal β-cell development and function.

## Discussion

The studies reviewed above collectively indicate that, while β-cells have low activity of catalase and glutathione peroxidase compared to other tissues (14, 16, 40), they maintain a robust antioxidant system primarily requiring the cytoplasmic thioredoxin reductase 1 (TXNRD1), thioredoxin 1 (TXN1), and peroxiredoxin 1 (PRDX1). NAPDH for this system is provided in the cytoplasm by glucose-6 phosphate dehydrogenase (G6PD). These conclusions are primarily based on depletion of the endogenous enzymes, but several other studies suggest that overexpression of thioredoxin or different peroxiredoxin isoforms grant β-cells additional protection against oxidative damage.

However, in addition to their roles in ROS detoxification, 2-Cys peroxiredoxins participate in hydrogen peroxide “redox relays” *via* the formation of mixed disulfide intermediates with other proteins (**Figure 1C**) (75). Here, peroxiredoxins are oxidized at the peroxidatic Cys by hydrogen peroxide, but instead of forming a disulfide bond by reacting with the resolving Cys, redox-sensitive cysteine residues on target proteins are oxidized, allowing peroxiredoxins to play a critical signaling role (**Figure 1D**). While hydrogen peroxide toxicity requires concentrations in the micromolar range, concentrations in the nanomolar range can promote signaling (4). Indeed, addition of low concentrations of hydrogen peroxide as a bolus increases basal insulin secretion, and addition of antioxidants catalase or *N*-acetyl-L-cysteine (NAC) blunts glucose- or KCl-stimulated insulin secretion in INS 832/12 cells (31). Similarly, addition of a vitamin E analog decreases glucose stimulated insulin secretion (GSIS) in rat islets (33), all suggesting that hydrogen peroxide promotes insulin secretion. Based on these data, one could imagine a potential signaling role for peroxiredoxins, potentially through a redox relay, in promoting GSIS.

**Figure 2:**
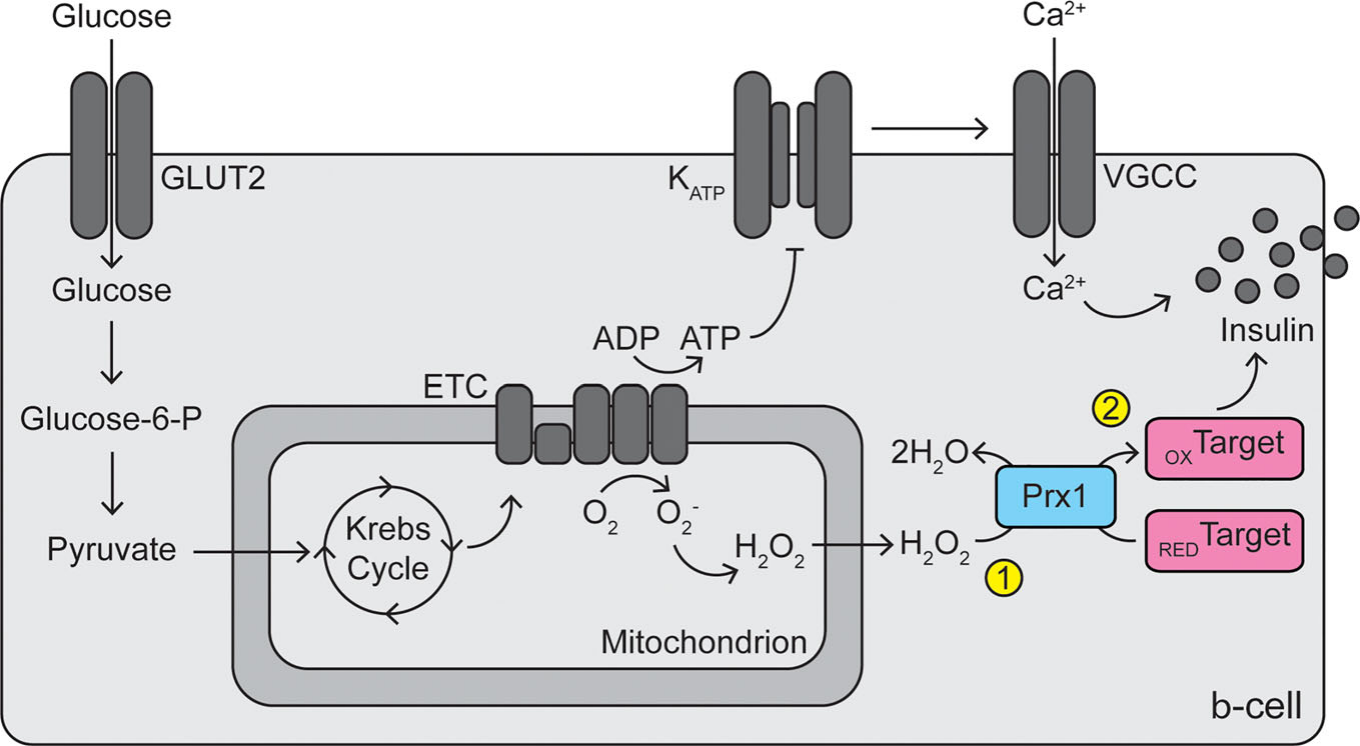
Dual roles of peroxiredoxins in β-cell protection and signaling. Reactive oxygen species (superoxide and hydrogen peroxide) are generated by electron leak during mitochondrial oxidative phosphorylation. Cytoplasmic peroxiredoxins (particularly PRDX1) play a primary antioxidant role in the β-cell by reducing hydrogen peroxide to water, protecting the cells from oxidative stress (1). In addition, peroxiredoxins may participate in a redox relay, utilizing hydrogen peroxide, to oxidize unknown target proteins, thus promoting glucose-stimulated insulin secretion (2). GLUT2, glucose transporter 2; Glucose-6-P, glucose-6-phosphate; ETC, electron transport chain; ADP, adenosine diphosphate; ATP, adenosine triphosphate; KATP, ATP-sensitive potassium channel; VGCC, voltage-gated calcium channel; Prx1, peroxiredoxin 1; RED, reduced; OX, oxidized.

While no studies have observed direct evidence for the existence of redox relays in β-cells, there is sufficient evidence that activity of peroxiredoxins, thioredoxin, or thioredoxin reductase promotes GSIS. Thioredoxin reductase inhibition by auranofin blunts GSIS, and addition of thioredoxin mimetics rescues the blunting, in INS 832/12 cells (56). Depletion of PRDX2 in *C. elegans* (76) or depletion of PRDX3 in RINm5F cells (68) decreases GSIS. Further, depletion of G6PD in MIN6 cells or mouse islets dampens GSIS (73), and global overexpression of G6PD increases β-cell electrical activity in response to high glucose (74). If hydrogen peroxide alone promoted GSIS, likely through direct oxidation of unknown target proteins, then inhibition or removal of thioredoxin reductase or peroxiredoxins, which should increase the intracellular hydrogen peroxide concentration, would promote insulin secretion. However, the opposite effect is observed, indicating that these enzymes are required for the signaling and suggesting the existence of a redox relay. Together, we speculate that cytoplasmic peroxiredoxins play a dual role in β-cells: as important protectors against oxidative damage induced by cytoplasmic hydrogen peroxide or peroxynitrite, and as signal transducers to stimulate GSIS (**Figure 2**). Future studies should be aimed at determining if mixed disulfide intermediates between peroxiredoxins and target proteins exist in β-cells, if these relays promote GSIS, and which target proteins are involved.

## Publisher’s Note

All claims expressed in this article are solely those of the authors and do not necessarily represent those of their affiliated organizations, or those of the publisher, the editors, and the reviewers. Any product that may be evaluated in this article, or claim that may be made by its manufacturer, is not guaranteed or endorsed by the publisher.

## Author Contributions

JS and JC wrote the manuscript. JS designed and created the Figures. JS and JC approved the submitted version. All authors contributed to the article and approved the submitted version.

## Funding

This work was supported by the National Institute of Diabetes and Digestive and Kidney Diseases grant DK-052194 (to JC), the National Institute of Allergy and Infectious Diseases grant AI-044458 (to JC), and by a gift from the Forest County Potawatomi Foundation. JS was supported by the National Heart, Lung, and Blood grant T32-HL134643.

## Conflict of Interest

The authors declare that the research was conducted in the absence of any commercial or financial relationships that could be construed as a potential conflict of interest.

## Publisher’s Note

All claims expressed in this article are solely those of the authors and do not necessarily represent those of their affiliated organizations, or those of the publisher, the editors and the reviewers. Any product that may be evaluated in this article, or claim that may be made by its manufacturer, is not guaranteed or endorsed by the publisher.
